# Relationship between Parental Socialization, Emotional Symptoms, and Academic Performance during Adolescence: The Influence of Parents’ and Teenagers’ Gender

**DOI:** 10.3390/ijerph16122231

**Published:** 2019-06-25

**Authors:** Paola Bully, Joana Jaureguizar, Elena Bernaras, Iratxe Redondo

**Affiliations:** 1 Paola Bully Consultoría Estadística y Metodológica, 48190 Sopuerta, Spain; 2 Developmental and Educational Psychology Department, Faculty of Education of Bilbao, University of the Basque Country, 48940 Lejona, Spain; joana.jauregizar@ehu.eus (J.J.); iratxe.redondo@ehu.eus (I.R.); 3 Developmental and Educational Psychology Department, Faculty of Education, Philosophy and Anthropology, University of the Basque Country, 20018 Bizkaia, Spain; elena.bernas@ehu.eus

**Keywords:** parental styles, parental warmth and control, teenagers, maladjustment, academic performance, gender

## Abstract

Scientific interest in students’ emotional and psychosocial experiences has been increasing in the last years due to their influence on students’ learning processes and academic performance. The present manuscript tries to go further in the study of the relationship between perceived parenting socialization and academic performance by analyzing not only their direct effects, but also by testing their indirect influence through other variables such as students’ psychological and school maladjustment, especially focusing on gender differences (both of students and parents). The sample comprised 823 students (416 males and 407 females) from the Basque Country (Spain), with ages ranging between 12 and 16 years (M = 13.7, SD = 1.2). Students completed a sociodemographic data form, the PARQ-Control questionnaire, and the BASC-S3 test. Teachers answered an ad hoc question on each student’s academic performance. The data showed that, both for males and females, the same structure of parent–teenager relationship predicted teenagers’ academic performance, via psychological and school maladjustment. However, the intensity of the relationship between parental acceptance and teenagers’ results in all the other factors differed depending on teenagers’ gender. Fathers’ influence was greater in males, and mothers’ influence was higher in females. This study is considered a starting point for a theoretical model predicting academic performance and psychological and school maladjustment among teenagers.

## 1. Introduction

One of the aspects that raise considerable concern in the educational community is adolescents’ school failure [1], given the negative effect that it has at this stage on their future, and on society in general.

According to the data, 10.6% of the people in Europe between 18 and 24 years drop out early from education-training (without completing the second stage of compulsory secondary education or following any other type of education-training). These data are even more worrying in Spain because, according to data from the Ministry of Science, Innovation and Universities [2], this dropout rate reaches 17.9% (7.0% of students abandon their studies in the first stage of secondary education, and 10.9% in the second stage). Furthermore, the number of adolescents who cannot reach the average level of academic performance and the pedagogical level expected for their age in Spain gradually increases throughout secondary education. Thus, one third of the students fail to complete secondary education, and more than 40% start the last year with added delay [3].

Therefore, it is important to study the possible factors associated with both school adjustment in general and academic performance in particular, taking into account the ecological perspective of adolescent development [4]. One of the aspects that requires special attention is the family micro-system. Despite the growing filial autonomy that occurs during adolescence [5], theory and empirical research have shown for decades a broad consensus regarding the crucial importance of parent–child relationships in children’s and adolescents’ development and adjustment [6,7,8,9], with effects that persist into adult life [10]. Beyond the conceptualization of parental interactions, which seem to depend on the context and the culture in which they are evaluated, the “domain-specific” models appear to be more flexible and situational because they provide more specificity in understanding parenting effects [11,12]. Specifically, the dimensions that have received the most studied attention are “warmth” and “control”.

Scientific evidence suggests, for example, that parental warmth and affection are related to better filial personal adjustment [13,14], higher self-esteem [15,16], self-confidence [17], and lower risk of depression and anxiety [18,19,20]. Furthermore, they are associated with greater attachment to parents and peers [21], lower peer-victimization levels [22], fewer violent, aggressive, or bullying relationships with peers [23], and, therefore, with better adjustment to the school context [24] and better academic performance [25]. In addition, it is considered that perceiving parental support may be a protective factor against the possible negative and stressful effect of school failure and other environmental factors [26]. On the contrary, low parental warmth/affection during adolescence is related to behavior problems, such as delinquency [27] or substance use [28], and to a worse academic performance [29,30].

Parental control is understood as the degree to which fathers and mothers regulate or manage their children’s behavior, for example, by insisting they obey rules or demands [31] known as “behavioral control” (following the distinction of behavioral and psychological control made by Schaefer, 1965). Research about this concept has obtained less consistent results [32], especially in different cultural contexts [14,16]. Some studies have found that greater parental control is related to adolescents’ better psychological adjustment [32,33,34,35], fewer antisocial and criminal activities [34,36], and a later sexual initiation [37], whereas others find a negative effect related to emotional [36,38,39,40] and behavioral problems [41]. Similarly, some studies have found a positive relationship between parental behavioral control and academic performance in European-American teenagers, but not in African-American ones [33], whereas others have found no association [35] or a negative association [25]. In Spain, the results of previous studies seem to confirm that children who perceive their families as not only affectionate, but also not very authoritative (under parental control) are the ones that obtain the best levels of psychological adjustment [14,42], with indicators such as greater emotional stability and a positive world view [42], fewer behavioral problems [43], lower substance use [28], and better academic performance [42]. These results are confirmed even when the parents themselves are the informants about their socializing styles with their children [44]. A possible explanation for this different role of control in Spanish families is the fact that these families have been described as horizontal collectivistic [45,46], with egalitarian rather than hierarchical relationships, so strict control does not have the positive connotation that it may have in other cultural contexts [42].

Therefore, interactions with parents not only influence adolescents’ perceptions and “internal” factors, but they also indirectly affect teenagers’ adjustment and adaptation to their environment, and specifically, to the school environment.

One consideration to bear in mind in the study of the relationship between parental socialization and filial results is parental gender. Parents’ gender seems to influence adolescents’ evaluation and interpretation of parental behavior, and, through this interpretation, their development. It has been proven, for example, that the meaning attributed to excessive control or a lack of warmth is not the same when they are attributed to fathers or mothers. Hence, some studies point out that maternal warmth-acceptance, but especially paternal warmth, are the factors with the greatest weight in the adolescents’ outcomes [6,25,35]. Other studies have found that, while the perception of a mother lacking in affection is negative for children’s self-concept, perceiving the father as not very affectionate seems to be more innocuous [35,47]. On the other hand, Chen et al. [48] found that only the father’s warmth predicted adolescents’ social and school achievement, whereas the mother’s warmth was a predictor of adolescents’ emotional adjustment. With respect to control, high parental control perception seems to favor the adolescent’s adjustment when exercised by the father, whereas when perceived in the mother, results are not as beneficial, even if both parents are perceived as affectionate and sensitive [47].

Finally, in addition to parents’ gender, adolescents’ gender may influence their assessment or interpretation of the relationships with their parents. For example, it is common for girls to score higher in the perception of parental support [49]. With respect to control, the few gender-sensitive existing studies show contradictory results; in some cases, they have found that parental monitoring of adolescent activities is more important among males than among females [50], whereas other studies find the opposite [51]. On the other hand, in academic aspects such as engagement, higher scores tend to correspond to females [52], who also show a greater sense of belonging to school [53], greater weight of academic sphere in their life satisfaction [54], and lower rate of academic failure [55].

In view of these results, in order to understand the way in which parental socialization relates to academic performance, we consider it necessary to study, in addition to its direct effects, the possible mediating effect of psychosocial and school adjustment, taking into account the role of both parents separately and the gender of the adolescents. These aspects, which have hardly been taken into account in previous research, constitute the cornerstone of our study.

## 2. Materials and Methods

### 2.1. Participants

An incidental sampling was carried out that concluded with the participation of 70 teachers and 823 high school students (50.5% males and 49.5% females) from the provinces of Vizcaya, Guipúzcoa, and Álava in the Basque Autonomous Community (Spain). The students’ age ranged between 12 and 16 years (M = 13.7, SD = 1.2). Participants came from both public and private schools (75.7% and 24.3%, respectively) and also from different backgrounds (28.2% rural and 71.8% urban). No relevant differences were found in the variables studied according to the contextual characteristics.

### 2.2. Measures

#### 2.2.1. Parental Acceptance-Rejection/Control Questionnaire (PARQ/Control)-Spanish Version

The PARQ/Control [31] assesses individual perceptions of parental warmth and control among children aged 7 to adolescence. In this study, two scales of this questionnaire were used: the Warmth scale [56] and the Control scale [31,57]. The Warmth scale evaluates adolescents’ perceptions of love and affection received from their parents (or from significant others), and includes their perceptions of the acceptance of their personality, the interest their parents show in their activities and well-being, enjoyment of free time together, and parents’ ability to comfort, console, praise, and embrace or express their love through words and actions. The Parental Control scale evaluates adolescents’ perceptions of behavioral control exercised by their parents or significant others (permissiveness or strict control). The Warmth and the Control scales consist of 20 and 13 items, respectively, and are scored on a 4-point Likert scale ranging from 1 Almost never true to 4 Almost always true. All the items must be answered separately referring to the mother and the father (or caretakers) in order to obtain separate results about the relationship with each figure. The fit indexes obtained in confirmatory factor analysis (CFA) were satisfactory (Warmth scale: χ^2^ = 750.70, *p* < 0.001, χ^2^/df = 4.41, comparative fit index (CFI) = 0.93, root mean square residual (RMSEA) = 0.06, 90% confidence interval (CI) (0.06, 0.07), standardized root mean square residual (SRMR) = 0.04; Control scale: χ^2^ = 118.95, *p* < 0.001, χ^2^/df = 4.40, CFI = 0.94, RMSEA = 0.06, 90% CI (0.05, 0.07)), SRMR = 0.04). The Cronbach alpha scores in this study for these two scales were high, that is, 0.94 for Warmth and 0.86 for Parental Control.

#### 2.2.2. Behavior Assessment System for Children and Adolescents (BASC) Basque Adaptation by Bernaras, Jaureguizar, Soroa, and Sarasa, in Its Self-Report Version for Adolescents Aged 12 to 18 (S3)

The S3 [58,59] is a self-report personality assessment inventory composed of 185 statements rated as True or False. In the present study, only two of its dimensions were explored: the Emotional Symptoms Index (ESI) and School Maladjustment (SM). The ESI is a global indicator of serious emotional disturbance, particularly of internalizing disorders. It is composed of four scales related to the Internalizing Problems factor (Social Stress, Anxiety, Depression, and Sense of Inadequacy) and two scales related to the Personal Adjustment factor (Self-esteem and Self-reliance). SM is a measure of generalized maladjustment at school (negative attitudes towards school staff and towards the educational system and process in general), which is often accompanied by academic difficulties. In this study, the questionnaire showed adequate psychometric properties (χ^2^ = 180.58, *p* < 0.001, χ^2^/df = 2.65, CFI = 0.80, RMSEA = 0.06, 90% CI (0.05, 0.07)), SRMR = 0.05). Cronbach’s alphas were 0.92 for ESI and 0.83 for SM.

#### 2.2.3. Academic Performance

Teachers reported the academic performance of all the students who participated in the study. Specifically, they had to compare each student’s academic level with the average performance of their classmates on a 5-point Likert scale, ranging from 1 (well below the class average) to 5 (well above the class average).

### 2.3. Procedure

Before contacting the schools, study procedures were approved by the Research Ethics Committee of the University of the Basque Country (Exp.M10_2018_185). Subsequently, in direct interviews with the school headmasters, the aims, procedures, and materials to be used in the study were explained. After a school headmaster had agreed to participate, the conditions were established for the students’ families to receive the informed consent. Those families that allowed their children to take part in the study sent the written informed consent back to the school (approximately 80% of the parents agreed to participate). The tests were administrated collectively. Students filled out the questionnaires during regular classes. A research team member read the instructions to complete the questionnaires out loud.

### 2.4. Statistical Analysis

First, the psychometric properties of the instruments were checked. For this purpose, we carried out a CFA and calculated the reliability and discrimination indexes. Then, we calculated descriptive statistics, mean differences, and correlational analyses.

Next, we tested the proposed empirical model, which included the maternal and paternal warmth and control dimensions as the antecedents, the ESI and SM dimensions as mediators, and the teachers’ perception of teenagers’ academic performance as the criterion variable. We used path analysis to test the mediation effects.

Lastly, a multi-group analysis of invariance was used to determine the moderation effect of teenagers’ gender, via structural equation modeling. Estimates were obtained using the maximum likelihood method. Absolute fit indexes (chi-square (χ^2^), χ^2^/df, standardized root mean square residual (SRMR)), relative fit indexes (incremental fit index (IFI)), and non-centrality fit indexes (comparative fit index (CFI), root mean square residual (RMSEA)) were used to assess model fit, as well as acceptance or rejection criteria based on the degree of fit described by [60]. Following the criteria proposed by Cheung and Rensvold [61], invariance is rejected if the value of the difference in CFI between the two nested models is higher than 0.01 in favor of the least strict model. SPSS v24.0 (IBM Corporation, Armonk, NY, USA) and AMOS v24.0 (IBM Corporation, Armonk, NY, USA) were used for all these analyses.

## 3. Results

### 3.1. Preliminary Analyses

Descriptive statistics are shown in Table 1. The results showed that males and females both agreed that their parents were more affectionate than controlling. Furthermore, they showed moderate levels of emotional symptomatology and school maladjustment, and their academic performance was about average.

Subsequently, a multivariate analysis of variance was conducted to test gender differences among the variables. The results indicated a significant effect of gender (F(7, 815) = 18.42, *p* < 0.001, η2p = 0.135). Post-hoc F-test analyses showed that differences in ESI (F(1, 821) = 17.07, *p* < 0.001), SM (F(1, 821) = 44.37, *p* < 0.001), and academic performance (F(1, 821) = 32.80, *p* < 0.001) were significant, as were differences in fathers’ warmth (F(1, 821) = 5.74, *p* < 0.017). However, the differences in mothers’ warmth, fathers’ control, and mothers’ control were not significant.

Bivariate correlations between the main variables were calculated to determine the relationships between the explanatory variables, and whether each variable correlated with the students’ academic performance before controlling for the effect of its relationship with other variables (Table 2).

As can be observed, fathers’ and mothers’ warmth and control were highly and directly correlated to each other. Furthermore, positive but moderate correlations were found between ESI and SM, whereas in the case of SM and academic performance, moderate, albeit negative, correlation coefficients were found. The magnitudes of the associations differed depending on gender; in most variable pairs, they were higher in the group of men.

### 3.2. Direct and Mediating Effects

**Figure 1:**
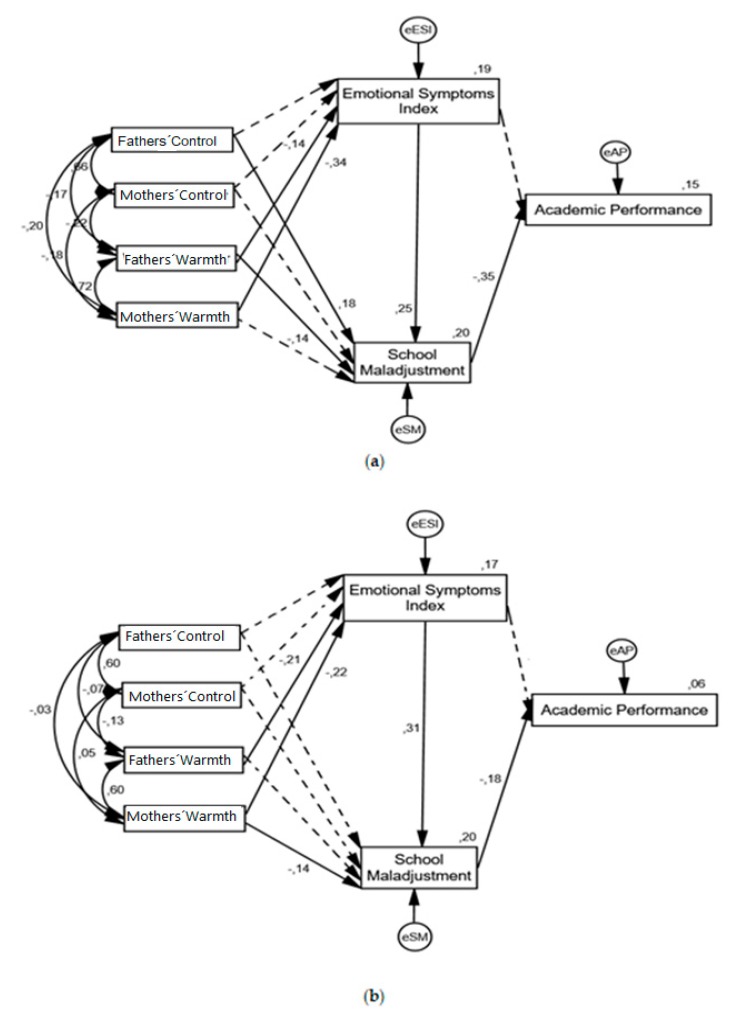
Standardized solution of the explanatory structural model (dashed lines indicate statistically non-significant relationships): (**a**) Males’ model; (**b**) Females’ model.

The model represents the mediating effect of SM and ESI in the relationship between parental socialization dimensions and academic performance (see Figure 1).

The skewness (Sk < 2) and kurtosis (K < 7) indexes indicated slight departures from the normal curve in all the variables (see Table 1). Nevertheless, Mardia’s multivariate kurtosis coefficients (34.90 for males, 20.66 for females) exceeded the critical ratio (11.82). Thus, in order to determine the influence of non-normality on the estimators, two types of analysis were performed [62,63]: one for the original sample using the maximum likelihood method, and the other for 1000 bootstrap samples, using the same method. A 95% confidence interval (CI) was set to evaluate corrected bias. The comparison of the results obtained by the two methods revealed no differences (*p* = 0.442 for males and *p* = 0.060 for females). Therefore, we examined the results of the analysis performed on the original sample.

When the theoretical model was applied to the entire sample, the goodness-of-fit indexes were as follows: χ^2^(4) = 12.04, *p* = 0.017, χ^2^/df = 3.01, with SRMR = 0.020, IFI = 0.994, CFI = 0.994, and RMSEA = 0.049, 95% CI (0.019, 0.083). Table 3 shows the different goodness-of-fit indexes of the model used for the two samples (males and females). In the case of males, both the chi-square inferential test and the other indexes presented satisfactory values (significance level associated with the statistic above 0.05, χ^2^/df lower than 3, IFI and CFI above 0.95, SRMR and RMSEA less than 0.05). The same occurred in the group of females. These results indicated that the model fits the two groups separately.

### 3.3. Multi-Group Analysis: The Moderator Effect of Teenagers’ Gender

The initial step in evaluating invariance requires both the same number of factors and of factor-load patterns in all groups. No equality restrictions are set on any of the parameters. Therefore, the same parameters that were estimated in the base model for each group separately were again calculated in this multi-group model. In methodological literature, this model is called the “configural model” (unconstrained model). The indexes obtained for this first multi-group invariance model revealed a non-significant chi-square value. As a result, the hypothesis of configural invariance was accepted, and it can be concluded that this model fits well in male and female samples when they are analyzed together (see Table 4). Metric invariance involves adding restrictions on regression coefficients to the base model (structural weights model). At this point, it is worth mentioning that the increase in chi-square was not statistically significant and there was a decrease of 0.001 in CFI. These data allow us to accept the metric model of invariance, and therefore, it can be concluded that the factorial weights were equivalent in the two subsamples. By adding intercept restrictions to this model, the increase in chi-square was statistically significant, and the increase in CFI (ΔCFI = 0.080) exceeded the cut-off point (0.01) proposed by Cheung and Rensvold [61]. This result was repeated in the following steps.

Based on these data, it can be concluded that, although the structure in the two groups may be considered equivalent, the relationship between parental socialization dimensions and teenagers’ psychological and school adjustment and academic performance cannot be considered invariant between males and females (Table 4).

In male students, the lower the parental warmth exercised by the father, the greater the presence of emotional symptomatology in the teenager, which leads to poorer school adjustment. Furthermore, higher school maladjustment leads to poorer academic performance. Only the father’s control and warmth were inversely related to school maladjustment, with school maladjustment being a mediating variable of academic performance. The model explained 15% of the variance of academic performance.

In female students, school maladaptation inversely mediated the relationship between the mother’s warmth and academic performance: the higher the level of maternal warmth, the lower school maladjustment, and the lower school maladjustment, the better was academic performance. However, both parents’ warmth was found to be negatively associated with the Emotional Symptoms Index (the greater the parents’ warmth, the lower the emotional symptomatology), which, in turn, was positively associated with school maladjustment, and school maladjustment was inversely related to academic performance. However, the explanatory capacity of the model in female students in this group was lower (R^2^ = 0.06).

No direct relationship was found between the parental socialization dimensions and academic performance or between emotional symptomatology and academic performance in either of the groups. The overall and indirect effects of the model are presented in Table 5.

## 4. Discussion

The purposes of this study were the following: (1) to analyze the relationships between parents’ warmth and control, teenagers’ psychological and school maladjustment, and academic performance as perceived by their teachers; (2) to determine the influence of the paternal and maternal roles separately; and (3) to explore the moderating effects of adolescents’ gender on these relationships.

The tested model presented a good fit to the data. First, a statistically significant, positive, and strong association can be observed between adolescents’ perception of their fathers’ and mothers’ warmth and the level of control, which seems to indicate a tendency to perceive coherent parental socialization styles between the parents. However, the adolescents’ outcomes derived from their parents’ educational practices were different.

Unlike findings in previous studies, such as those of [25] or [48], and according to the model we tested in this study, the perceived parental socialization dimensions did not contribute directly and significantly to teenagers’ academic performance. However, perceived parental warmth (fathers’ and mothers’) contributed inversely and significantly to the presence of emotional symptoms (comprising the dimensions of social stress, anxiety, depression, sense of inadequacy, self-esteem, and self-reliance) in teenagers, as in previous studies [13,19,20]. Furthermore, emotional symptomatology did not contribute directly to academic performance; nevertheless, it did contribute to school maladjustment, and this, in turn, had a negative effect on academic performance. Although some studies in line with the ”adjustment erosion model” show that internalizing and/or externalizing difficulties lead to later academic difficulties [64], some authors have detected no significant links between internalizing or externalizing problems and later academic achievement [65]. The authors in the last study hypothesized that some other variables that were not investigated may contribute to preventing problem behaviors from interfering with student learning, such as the teacher’s attitude or capability to manage the students, which suggests the influence of an unknown mediating factor. In our study, emotional symptomatology itself may not have a direct and negative effect on academic performance, although once emotional symptomatology has exteriorized as or has led to negative attitudes towards school, such negative attitudes will probably contribute to a poorer academic performance.

In the case of school maladjustment (negative attitude towards school and teachers and sensation-seeking behavior), only the control and warmth exercised by the father showed a statistically significant effect; higher control and lower warmth were associated with greater maladjustment. A possible explanation for this may be that the instrumental role traditionally associated with the father takes on greater relevance in adolescence [9]. In this sense, and according to our observations in this study, some authors point out that, when behavioral control is too strict, the consequences for academic performance may be negative, regardless of the student’s intellectual level [66]. As expected, school maladjustment has a significant and inverse impact on academic performance.

Regarding gender differences, the results indicate that the model fits male and female groups, and that the same structure, albeit with different parameters, can be accepted, regardless of the teenagers’ gender. One of the main differences is observed in the control dimension. In the case of males, an effect of the control exercised by the father was observed, namely a negative effect on school adjustment, which was not observed in females. The negative effect of control supports the hypothesis that, within the cultural context of parental control, it does not play a protective role against their sons’ problems of school adjustment. The fact that these same results are not found in daughters could be due to the fact that in both groups, the educational practices of the same-sex parent seem to be more important, although the differences were not statistically significant. In addition, emotional symptomatology had a higher association with school maladjustment in the case of females. A tentative explanation for this fact might be the greater prevalence of emotional symptomatology in girls, which has been observed in many studies. In fact, a vast number of studies have reported higher levels of anxiety and depression among girls when compared to boys [67,68,69,70,71]; specifically, higher mean scores on the internalizing factor of the BASC-S3 have also been found [59].

Lastly, the association between school maladjustment and academic performance was lower among females than among males. This could be explained due to the gender differences that usually appear with regard to these factors. In fact, studies tend to report both higher rates of school failure [2,55] and school maladjustment in adolescent boys compared to girls [72,73], with the boys showing a higher tendency to sensation seeking and presenting a negative attitude towards school [59]. These findings are in line with previous studies that indicate that daughters and sons show a different perception of paternal and maternal education [15], such that this interpretation could be conditioning their results in terms of psychosocial and educational adaptation (adjustment to the school environment and performance).

This study presents some strengths and limitations that should be mentioned. The main limitation has to do with the cross-sectional nature of the study, which, being strict, precludes establishing causal relationships among the studied variables. In addition, the two-stage sampling that was carried out (initially, random, but later on incidental) demands caution when generalizing the findings of this study.

Finally, although students and teachers both participated in the present study, future studies should also include parents, mainly to contrast their perceptions with adolescents’ perceptions of parental warmth and control, although it should be noted that previous studies have indicated that children provide reliable results about their parents’ parenting socialization styles [74,75,76]. Similarly, it would be interesting to test whether the children’s age also influences the perception of parentality and, consequently, the children’s results. All these issues have yet to be analyzed in future studies.

## 5. Conclusions

The results lead to the following conclusions and practical implications:

(1) Warmth/affection are considerably more relevant than control in adolescents’ positive development, which is in line with the results of previous studies in the Spanish context that conclude that “the key to effective socialization is parental warmth and involvement (all parenting styles with low levels of parental warmth tend to perform worse)” [42], (p. 123).

(2) Maternal and paternal affection contribute similarly and favor social [21], personal [77], and academic adjustment [78]. Therefore, awareness-raising programs or parent schools should emphasize the importance of the role of both parents (and not just the mother, as is most frequently highlighted in the scientific literature), especially in terms of displays of affection and warmth for adolescents’ psychosocial adjustment and academic performance.

(3) The control and warmth exercised by the father affects the boys’ results, with control being harmful and warmth being beneficial in the case of school adjustment. Therefore, it is recommended to increase warmth in child–parent relationships and to avoid excessive control, replacing it with close and frank communication between parents and children and some kind of inductive discipline and age-appropriate supervision [15].

(4) Paternal practices are far from being innocuous, reaffirming the importance of the involvement of both parents in adolescents’ education.

(5) Differences were found in the influence of parental socialization dimensions related to teenagers´ gender.

(6) The educational practices of the same-sex parent seem to have a greater importance. These results show that we should help parents to become aware of their role as figures with which their adolescent children can identify. This issue is central because, in all cases, the socialization practices of the same-sex parent are shown to have a greater effect. This is not surprising, as adolescence is a stage in which the construction of personal and sexual identity is extremely relevant.

## Figures and Tables

**Table 1:** Descriptive statistics of study variables for males, females, and total sample.

Variables	Males (*n* = 417)				Females (*n* = 406)				Total (*n* = 823)			
	M	SD	Sk	K	M	SD	Sk	K	M	SD	Sk	K
FW	67.66	9.80	−1.32	2.13	65.85	11.72	−1.50	2.51	66.77	10.82	−1.48	2.65
MW	69.97	9.13	−1.88	5.08	70.02	8.84	−1.83	4.71	70.00	8.98	−1.86	4.88
FC	29.48	5.98	0.29	0.35	29.83	6.42	0.32	0.01	29.65	6.20	0.31	0.18
MC	27.87	5.90	0.55	0.83	27.87	6.43	0.65	0.53	27.87	6.16	0.61	0.67
ESI	289.32	46.27	1.67	3.25	303.21	50.10	1.26	1.69	296.17	48.67	1.43	2.26
SM	158.89	25.14	0.42	−0.40	147.93	21.89	0.33	−0.65	153.48	24.21	0.44	−0.30
AP	3.03	1.08	0.02	−0.63	3.44	1.00	−0.13	−0.72	3.23	1.06	−0.09	−0.66

Note: FW = Fathers’ Warmth, MW = Mothers’ Warmth, FC = Fathers’ Control, MC = Mothers’ Control, SM = School Maladjustment, ESI = Emotional Symptoms Index, AP = Academic Performance, Sk = Skewness, K = Kurtosis.

**Table 2:** Correlations for males, females, and total sample with study variables.

Variables		FW	MW	FC	MC	SM	ESI
Mothers’ Warmth	Males	0.718 **					
	Females	0.596 **					
	Total	0.647 **					
Fathers’ Control	Males	−0.166 **	−0.202 **				
	Females	−0.072	−0.032				
	Total	−0.115 **	−0.116 **				
Mothers’ Control	Males	−0.220 **	−0.182 **	0.660 **			
	Females	−0.133 **	0.055	0.603 **			
	Total	−0.170 **	−0.062	0.629 **			
School Maladjustment	Males	−0.328 **	−0.343 **	0.159 **	0.082		
	Females	−0.295 **	−0.310 **	0.091	0.057		
	Total	−0.280 **	−0.320 **	0.116 **	0.068		
Emotional Symptoms Index	Males	−0.354 **	−0.407 **	−0.042	−0.012	0.344 **	
	Females	−0.330 **	−0.355 **	−0.100*	−0.121 *	0.380 **	
	Total	−0.347 **	−0.376 **	−0.068	−0.069	0.314 **	
Academic Performance	Males	−0.090	−0.160 **	0.047	0.001	−0.379 **	−0.215 **
	Females	−0.095	−0.163 **	−0.118 *	−0.093	−0.221 **	−0.164 **
	Total	−0.073 *	−0.159 **	−0.039	−0.045	−0.340 **	−0.156 **

Note: FW = Fathers’ Warmth, MW = Mothers’ Warmth, FC = Fathers’ Control, MC = Mothers’ Control, SM = School Maladjustment, ESI = Emotional Symptoms Index, * *p* < 0.05, ** *p* < 0.01

**Table 3:** Fit indexes of model across gender.

Group	χ^2^	df	*p*	χ^2^/df	SRMR	IFI	CFI	RMSEA (95% CI)
Males	3.76	4	0.440	0.94	0.012	0.999	0.999	0.000 (0.000, 0.070)
Females	10.60	4	0.031	2.65	0.032	0.989	0.988	0.044 (0.017, 0.112)

**Table 4:** Fit indexes for multi-group analysis of invariance across gender.

Model	χ^2^	df	*p*	Δχ^2^	*p* Δχ^2^	CFI	ΔCFI
Unconstrained	14.36	8	0.073	ne	ne	0.995	ne
Structural weights	24.98	19	0.161	10.66	0.475	0.996	−0.001
Structural intercepts	136.36	22	0.000	111.38	<0.001	0.916	0.080
Structural means	148.50	26	0.000	12.14	0.016	0.910	0.006
Structural covariances	213.79	36	0.000	65.28	<0.001	0.869	0.041
Structural residuals	225.79	39	0.000	11.99	0.007	0.863	0.006

Note: ne = not estimable.

**Table 5:** Standardized total and indirect effects of the model by gender.

Variables	Total Effects						Indirect Effects					
	MC	FC	MW	FW	ESI	SM	MC	FC	MW	FW	ESI	SM
Males												
ESI	−0.027	−0.115	−0.337	−0.137	0.000	0.000	0.000	0.000	0.000	0.000	0.000	0.000
SM	−0.094	0.151	−0.202	−0.179	0.252	0.000	−0.007	−0.029	−0.085	−0.035	0.000	0.000
AP	0.035	−0.041	0.102	0.075	−0.183	−0.346	0.035	−0.041	0.102	0.075	−0.087	0.000
Females												
ESI	−0.099	−0.063	−0.224	−0.214	0.000	0.000	0.000	0.000	0.000	0.000	0.000	0.000
SM	0.006	0.069	−0.210	−0.164	0.311	0.000	−0.031	−0.020	−0.069	−0.066	0.000	0.000
AP	0.008	−0.007	0.060	0.050	−0.152	−0.185	0.008	−0.007	0.060	0.050	−0.057	0.000

Note: FW = Fathers’ Warmth, MW = Mothers’ Warmth, FC = Fathers’ Control, MC = Mothers’ Control, ESI = Emotional Symptoms Index, SM = School Maladjustment.
